# Twelve-month findings of the MOVE Frankston randomised controlled trial of interventions to increase recreation facility usage and physical activity among adults

**DOI:** 10.1371/journal.pone.0254216

**Published:** 2021-07-23

**Authors:** Ben J. Smith, Ruth Mackenzie-Stewart, Fiona J. Newton, Karine E. Manera, Tilahun N. Haregu, Adrian Bauman, Robert J. Donovan, Ajay Mahal, Michael T. Ewing, Joshua D. Newton

**Affiliations:** 1 School of Public Health, Level 6, The Charles Perkins Centre, University of Sydney, Sydney, New South Wales, Australia; 2 School of Public Health and Preventive Medicine, Monash University, Melbourne, Victoria, Australia; 3 School of Psychology and Public Health, LaTrobe University, Bundoora, Victoria, Australia; 4 Department of Marketing, Monash Business School, Monash University, Frankston, Victoria, Australia; 5 Nossal Institute for Global Health, Melbourne School of Population and Global Health University of Melbourne, Melbourne, Victoria, Australia; 6 School of Human Sciences, University of Western Australia, Perth, Western Australia, Australia; 7 Department of Marketing, Deakin Business School, Deakin University, Geelong, Victoria, Australia; Prince Sattam Bin Abdulaziz University, College of Applied Medical Sciences, SAUDI ARABIA

## Abstract

Substantial cross-sectional evidence and limited longitudinal research indicates that the availability of recreational facilities (e.g., parks, fitness centres) is associated with physical activity participation. However, few intervention trials have investigated how recreational infrastructure can be used to reduce inactivity levels in communities. The MOVE Frankston study aimed to assess the impact of low intensity strategies to promote use of a multi-purpose leisure and aquatic centre in a socioeconomically diverse, metropolitan community. This randomised controlled trial of two years’ duration compared public awareness raising (control condition) with two interventions: mailed information about the centre and a free entry pass (I-O); and this minimal intervention supplemented by customer relations management support through telephone contact, mailed promotional materials and additional incentives (I+S). Participants (n = 1320) were inactive adults living in the City of Frankston, Melbourne Australia. There were 928 people (70.3%) followed up at 12 months (61.2% female, 52% ≥55 yrs). Compared with controls, attendance at the Centre once or more was higher in both the I-O (OR 1.79, 95% CI 1.28–2.50) and I+S groups (OR 1.46, 95% CI 1.03–2.07). The proportion of people using the centre weekly did not differ by group. The odds of being in contemplation or preparation to use the Centre were higher in both the I-O (OR 1.76, 95% CI 1.28–2.42) and I+S groups (OR 1.48, 95% CI 1.07–2.06). Total physical activity and related social and cognitive factors did not differ between the groups. The findings show that the low intensity promotional strategies prompted occasional attendance and increased readiness to use this recreational facility, a level of behaviour change unlikely to reduce non-communicable disease risk. It is recommended that more frequent customer relations contact, and involvement of healthcare providers, be tested as strategies to encourage inactive adults to take up physical activity opportunities at recreational facilities of this type.

## Introduction

Physical inactivity is the fourth leading cause of global deaths due to non-communicable diseases (NCDs) [1], and is strongly related to the risk of heart disease, stroke, type 2 diabetes, and colon cancer, among a range of other conditions [2]. It is therefore concerning that national reports show only 50 percent of Australian adults are sufficiently active [3], with inactivity more common among women than men, and in those aged 65 years and over. A number of policies and programs have been implemented over the past few decades to address the public health threat posed by physical inactivity, with modification of the built environment and provision of local opportunities for physical activity being recognised as important elements of a whole-of-systems approach to physical activity promotion [4, 5].

The built environment incorporates land use patterns, transportation systems and urban planning characteristics [6]. Certain features of the built environment, including sidewalks, trails, parks, public transport, and recreation and exercise facilities, are associated with increased physical activity [4, 7]. By extension, the development of community facilities such as multi-purpose leisure, aquatic and fitness centres may have potential to deliver public health benefits for the surrounding community. This is particularly the case in Australia, where data suggest fitness and gym activities are the most commonly reported sport or recreation after walking [8].

Despite their potential, there is limited information on the impacts of community recreation facilities upon physical activity in surrounding populations. Systematic reviews of the literature examining the relationship between recreation facilities and physical activity have found that most studies have used cross-sectional designs, and have highlighted a need for longitudinal and interventional studies to generate better evidence of causal relationships [9, 10]. Of particular interest to health policy makers and service providers are the practical and scalable strategies that can increase usage of these facilities to increase physical activity, especially among the insufficiently active who are at elevated risk of diabetes, cardiovascular disease, musculoskeletal disorders and other chronic conditions.

Among the few strategies that have been tested to increase the use of leisure centres, those that have shown most promise so far have offered free entry during certain times or free membership to inactive individuals [11, 12]. Given the high costs entailed in these approaches, they have uncertain feasibility and sustainability for centre operators, and for local and provincial governments. Little is known about the effectiveness of less costly marketing and customer-relationship management (CRM) strategies, in encouraging the use of these facilities for physical activity promotion and public health. These strategies have been used widely in commercial and, to a lesser extent government service provision, to improve the value experience for consumers and build relationships and loyalty through various methods of contact, communication and reinforcement (e.g., telephone, mail, internet) [13, 14].

In order to address this evidence gap, the Monitoring and Observing the Value of Exercise (MOVE) trial aimed to assess the impact of low intensity interventions upon leisure and aquatic facility attendance and physical activity participation among adults who are typically inactive. These interventions were trialled because they could be readily integrated into existing customer relationship management systems and did not require ongoing fiscal outlays in the form of free entry vouchers. It was hypothesised that simple incentives could prompt use of the facility by local residents, and more frequent customer contact (by phone and mail) would lead to higher levels of attendance and greater improvements in overall physical activity. The findings from this trial could provide a model for strategies and partnerships to leverage the benefits of existing recreational facilities to tackle physical inactivity and the associated burden of NCDs in communities.

## Materials and methods

The MOVE Frankston trial methods and interventions have been reported in the study protocol [15], and are summarised briefly here.

### Study design

A community-based RCT was conducted over two years (2014–2016) in the City of Frankston, a socioeconomically diverse municipality in outer metropolitan Melbourne, Australia (estimated population 137,790). This had ethical approval from the Monash University Human Research Ethics Committee (Project IDs: CF14/1148–2014000497 and CF14/2059–2014001074), and oral consent was required from participants prior to their enrollment into the study. The study was registered with the Australian New Zealand Clinical Trials Registry (Trial ID: ACTRN12615000012572). Because of the necessity to commence participant recruitment and baseline measurement before the Peninsula and Aquatic and Recreation Centre (PARC) was opened to the public, the registration of this trial was not formalised until after the field work had begun.

### Participants and recruitment

Study participants were adults aged 18–70 years who resided in the City of Frankston. Eligibility requirements were that individuals were typically inactive, i.e., reported less than five occasions of 30 minutes or more of physical activity per week to a validated assessment question [16], and did not attend a leisure or exercise facility three or more times per week. Exclusion criteria were an inability to walk independently, poor English proficiency, lack of telephone and postal means of communication, and having already purchased an annual membership of PARC before the study commenced.

The primary method of recruitment was by telephone calls to a random sample of persons listed in the Electronic White Pages directory for the City of Frankston area. Within household selection of participants was undertaken by computer generated random ordering of persons in the eligible age range. If the first selected person did not meet the screening criteria, the next listed individual in the household was screened until an eligible individual was identified. A small proportion of recruitment (5%) was undertaken by means of face-to-face invitations to people at community venues (e.g., shopping centres) in selected in socioeconomically disadvantaged suburbs. Sample size calculations showed that 939 participants would need to be recruited in order to detect a 10% difference (using a two-tailed test, α = 0.05, power = 0.80) [17] between each of the intervention conditions in weekly PARC attendance, assuming that 10% of those in the control group would achieve this outcome. In order to allow for attrition over the two year duration of the trial, which was considered may be higher in the control group (up to 40%) than the two intervention groups (up to 25%), the total sample size of 1300 was sought.

### Interventions

PARC is centrally located adjacent to the Frankston City Central Business District and was first opened in September of 2014, with an establishment cost of AUD$49.7 m. The Centre offers multiple leisure and exercise facilities, including: a 50-metre indoor lap pool; learn to swim pools; warm water pool and aquatic playground; gymnasium; group exercise rooms; and spa, sauna and wellness therapies centre.

Following recruitment and baseline measurement, participants were randomised on an individual basis using computer-generated number assignment, followed by sequential ordering and allocation to either the control group or one of the intervention groups. Random sequence generation was completed by BJS, and group allocation was implemented by JDN.

The control condition comprised the general community-wide promotion of PARC prior to its opening, through articles and advertising in local newspapers, letters to residents sent by the Frankston City Council, and public billboards. The interventions were designed using insights from Rogers Diffusion and Innovations model [18], social marketing [19] and customer relationships methods [14]. A minimal incentive only (I-O) intervention comprised a mailed information pack about PARC and a voucher for one free trial visit to use the swimming pools or gymnasium at the Centre. An incentive plus customer relations support (I+S) intervention included these basic elements in combination with a follow-up telephone call in the first 6 months to encourage trialling of the Centre, mailed newsletters on 4 occasions per year, and PARC-branded customer relations materials (birthday, Christmas and post cards), mailed several times each year.

### Measurement

The primary outcomes reported here are PARC attendance and physical activity participation. PARC attendance was measured by asking participants whether they had used the Centre in the past 12 months, and if so whether this was <once per month, 1–2 times per month, 1–2 times per week, or ≥3 times per week. Physical activity participation was measured by the Exercise Recreation and Sport Survey (ERASS), which assesses the frequency and duration of organised and non-organised leisure activities in the past two weeks and has concurrent validity in relation to established population measures of total physical activity [20]. Secondary outcomes assessed were stage of readiness to attend PARC, which was measured using an adapted version of a five-item stage of change for exercise scale developed by Marcus et al. and shown to have construct validity [21], as well as measures of social and cognitive determinants of physical activity. These included measures of: intentions and action planning, adapted from scales reported by Shuz et al. to have construct validity in relation to physical activity [22]; attitude and subjective norms concerning physical activity, developed by Ajzen [23]; self-efficacy to undertake physical activity, adapted from a scale developed by Armitage et al [24]; and anticipated regret concerning not undertaking physical activity, using items reported by Abraham and Sheeran to have internal reliability and construct validity [25]. Descriptive information collected about study participants included: gender; age; household composition; educational attainment; occupation; household income; language spoken; residential proximity to PARC (determined by their home address); and chronic disease status, measured by items pertaining to major conditions (e.g., heart disease, diabetes, arthritis) in the Functional Comorbidity Index [26].

All measurements were undertaken by computer assisted telephone interview. Baseline measures were collected at the time of recruitment, which occurred during the 6-week period leading up to the opening of PARC (August-September, 2014), while follow-up measurements were conducted 12 months later (September-October, 2015). An independent team of trained telephone interviews collected baseline and follow-up measures, and to ensure blinding they were only provided the name and contact details of study participants, with no disclosure of group allocation.

### Statistical analysis

Analysis was undertaken by intention-to-treat, including only those with complete follow-up data. To assess primary outcomes, the proportion of participants who attended PARC at least once over 12 months, ≥ monthly, or ≥ weekly were calculated. Using data from ERASS, total minutes of moderate− and vigorous−intensity physical activity (MVPA) in the most recent two weeks were calculated, with participants subsequently classified as very inactive (<20 min per week), low active (20–149 min per week), or sufficiently active (≥150 min per week). The proportions of participants who progressed across these categories from baseline to 12 months and who achieved a sufficient level of physical activity were then determined. In the analysis of secondary outcomes the responses to the stage of readiness to attend PARC measure were used to classify participants into precontemplation (no intention), contemplation/preparation (intention to attend in the next month, or six months), or action/maintenance stages (attending in the recent six months, or for longer than six months). In addition, the measures of social and cognitive influences upon physical activity, including intentions, attitudes, action planning, self-efficacy, subjective norm, and anticipated regret, were dichotomised (agree vs neutral/disagree) and the proportions of participants with each of these cognitive characteristics were calculated.

A minimum level of statistical significance of p<0.05 was adopted in analysis. Once it was ascertained that there were no significant differences in terms of demographic characteristics across the trial groups, differences in the outcomes were assessed using univariable logistic regression. To explore moderation of intervention effects, the comparison of group differences was stratified by gender. All analyses were undertaken using the IBM SPSS 24 software package.

## Results

**Fig 1:**
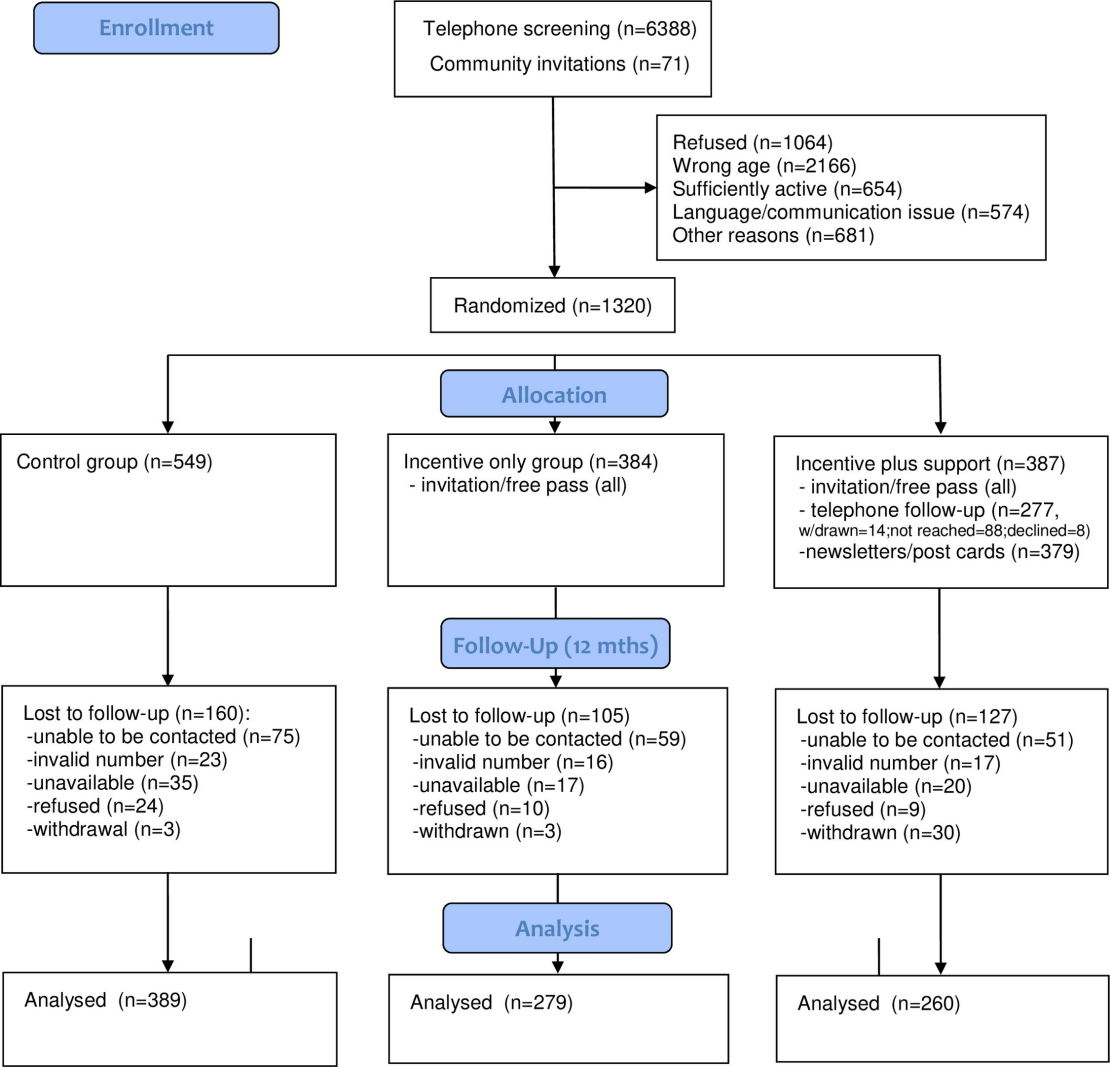
CONSORT flow diagram for MOVE Frankston study.

Of those residents who were contacted by telephone (N = 6388) or recruited through community venues in selected areas (N = 71), 1320 were enrolled and randomised to one of the trial groups. In all, 928 (70.3%) of the 1320 enrolled participants were successfully followed up at 12 months and were included in analysis. The major reasons for exclusion during recruitment or loss to follow-up are shown in CONSORT flow diagram for the study (Fig 1).

As shown in Table 1, around 60% were female and approximately half were aged 55 years or older. While over half were in full- or part-time employment, a substantial fraction belonged to the ‘other’ employment category (mostly retired). Around one-quarter had attained a university qualification and over one-third were educated up to a high school level only. Most did not have children aged 18 years or below living at home and the distribution of yearly household income was approximately evenly divided between those receiving <AUD$40,000, AUD$40–79,999, and ≥AUD$80,000. Approximately three-quarters of all participants had one or more chronic health conditions. A very small fraction spoke a language other than English at home. At baseline about one-third of participants reported <20 mins/week of MVPA in the past two weeks, while just under 40% reported 20–149 mins/week. Approximately three-in-five expressed an intention (i.e., were in the contemplation stage) to use the PARC facility. While there were no significant differences in demographic characteristics, physical activity or readiness to use PARC across the trial groups, those completing the 12-month follow-up measures were more likely to be aged ≥55 years, not in paid employment, to have a household income <AUD$40,000, and to usually speak English at home, than non-completers.

**Table 1:** Characteristics of participants at 12 months follow-up.

Characteristic	Control (N = 389)	Incentive only (N = 279)	Incentive+support (N = 260)	Total (N = 928)
	n (%)	n (%)	n (%)	n (%)
**Gender**				
Female	238 (61.2)	169 (60.6)	161 (61.9)	568 (61.2)
Male	151 (38.8)	110 (39.4)	99 (38.1)	360 (38.8)
**Age group (years)** ^**a**^^,^^**b**^				
18–34	44 (11.3)	26 (9.3)	18 (6.9)	88 (9.5)
35–54	144 (37.1)	104 (37.3)	109 (41.9)	357 (38.5)
≥55	200 (51.5)	139 (53.4)	133 (51.2)	482 (52.0)
**Employment status**^**b**^				
Full-time	120 (30.8)	89 (31.9)	81 (31.2)	290 (31.3)
Part-time	102 (26.2)	51 (18.3)	64 (24.6)	217 (23.4)
Other	167 (42.9)	139 (49.8)	115 (44.2)	421 (45.4)
**Education**^**a**^				
High school or less	115 (30.2)	109 (40.2)	97 (37.9)	321 (35.4)
Vocational qualification	160 (42.0)	89 (32.8)	96 (37.5)	345 (38.0)
University degree	106 (27.8)	73 (26.9)	63 (24.6)	242 (26.7)
**Children in household**				
None	240 (61.7)	174 (62.4)	162 (62.3)	576 (62.1)
≥1	149 (38.3)	105 (37.6)	98 (37.7)	352 (37.9)
**Household income (AUD)**^**a**^^,^^**b**^				
≤39,999	97 (28.3)	72 (29.8)	63 (27.5)	232 (28.5)
40,000–79,999	131 (38.2)	91 (37.6)	87 (38.0)	309 (38.0)
≥80,000	115 (33.5)	79 (32.6)	79 (34.5)	273 (33.5)
**Main language**^**a**^^,^^**b**^				
English	368 (98.1)	261 (98.9)	248 (98.0)	877 (98.3)
Other	7 (1.9)	3 (1.1)	5 (2.0)	15 (1.7)
**Chronic conditions**				
None	99 (25.4)	77 (27.6)	60 (23.1)	236 (25.4)
1	102 (26.2)	63 (22.6)	60 (23.1)	225 (24.2)
≥2	188 (48.3)	139 (49.8)	140 (53.8)	467 (50.3)
**Physical activity (past 2 wks)**				
<20 min/wk	131 (33.7)	91 (32.6)	98 (37.7)	320 (34.5)
20–149 min/wk	157 (40.4)	115 (41.2)	96 (36.9)	338 (39.7)
150+ mins/wk	101 (26.0)	73 (26.2)	66 (25.4)	240 (25.9)
**Stage of readiness for PARC**				
Precontemplation	115 (29.6)	99 (35.5)	89 (34.2)	303 (32.7)
Contemplation	241 (62.0)	157 (56.3)	156 (60.0)	554 (59.7)
Don’t know	33 (8.5)	23 (8.2)	15 (5.8)	71 (7.7)

^a^Data missing for participants: Age group, N = 1; Education, N = 20; Household income, N = 114; Main language, N = 36.

^b^Difference with those lost to follow-up (p<0.05).

### Usage of PARC

As shown in Table 2, higher proportions of study participants in the I-O (37.3%) and I+S groups (32.7%) than in the control group (24.6%) reported attending PARC once or more, with the odds ratios (OR) for this outcome being 1.79 (95% confidence interval (CI), 1.28–2.50) and 1.46 (95% CI 1.03,-2.07) in each of the intervention groups, respectively. Around 10% of each intervention group reported using the facility monthly, and just under 7% reported using it weekly, with these attendance levels not significantly different to those in the control group. Although more participants in the I+S group had become members of PARC, this difference did not reach significance.

**Table 2:** PARC attendance and PARC membership after 12 months.

Group	≥1 time in 12 months		≥1 Monthly		≥1 Weekly		Membership	
	n (%)	OR (95%CI)	n (%)	OR (95%CI)	n (%)	OR (95%CI)	n (%)	OR (95%CI)
**All (N = 928)**								
Control	97 (24.9)	1.00	34 (8.7)	1.00	18 (4.6)	1.00	16 (4.1)	1.00
Incentive only	104 (37.3)	1.79 (1.28,2.50)**	29 (10.4)	1.21 (0.72,2.04)	18 (6.5)	1.42 (0.73,2.78)	12 (4.3)	1.05 (0.49,2.25)
Incentive+support	85 (32.7)	1.46 (1.03,2.07)*	27 (10.4)	1.21 (0.71,2.06)	18 (6.9)	1.53 (0.78,3.01)	18 (6.9)	1.73 (0.87,3.47)
**Men (N = 360)**								
Control	25 (16.6)	1.00	8 (5.3)	1.00	5 (3.3)	1.00	4 (2.6)	1.00
Incentive only	39 (35.5)	2.77 (1.55,4.95)**	8 (7.3)	1.40 (0.51,3.86)	5 (4.5)	1.39 (0.39,4.93)	5 (4.5)	1.75 (0.46,6.67)
Incentive+support	24 (24.2)	1.61 (0.86,3.03)	7 (7.1)	1.36 (0.48,3.88)	3 (3.0)	0.91 (0.21,3.91)	3 (3.0)	1.15 (0.25,5.25)
**Women (N = 568)**								
Control	72 (30.3)	1.00	26 (10.9)	1.00	13 (5.5)	1.00	12 (5.0)	1.00
Incentive only	65 (38.5)	1.44 (0.95,2.18)	21 (12.4)	1.16 (0.63,2.13)	13 (7.7)	1.44 (0.65,3.20)	7 (4.1)	0.81 (0.31,2.11)
Incentive+support	61 (37.9)	1.41 (0.92,2.14)	20 (12.4)	1.16 (0.62,2.15)	15 (9.3)	1.78 (0.82,3.85)	15 (9.3)	1.94 (0.88,4.25)

*p<0.05;

**p<0.001.

Gender stratification showed that men in the I-O group were more likely than those in the control group to report attending PARC at least once (OR 2.77, 95% CI 1.55–4.95), whereas those in the I+S group were not. The other PARC attendance outcomes among men were similar across the intervention groups and not significantly higher than those in the control group. This pattern was also found among women, with no significant differences between the trial groups across measures of PARC attendance.

### Stage of readiness to use PARC

Table 3 shows that after 12 months participants in the I-O and I+S groups were significantly less likely than those in the control group to be in the precontemplation stage for PARC attendance, with odds ratios of 0.56 (0.41–0.77) and 0.62 (0.45–0.85), respectively. Further, relative to the control group, those in the I-O (OR 1.76, 95% CI 1.28–2.42) and I+S groups (OR 1.48, 95% CI 1.07–2.06) were more likely to be at the contemplation/preparation stages of readiness to attend PARC. There was no difference in the proportion of each group in the action/maintenance stages of PARC usage.

**Table 3:** Stage of readiness to use PARC at 12 months.

Group	Precontemplation		Contemplation/ Preparation		Action/ Maintenance	
	n (%)	OR (95%CI)	n (%)	OR (95%CI)	n (%)	OR (95%CI)
**All (N = 928)**						
Control	250 (64.3)	1.00	118 (30.3)	1.00	21 (5.4)	1.00
Incentive only	140 (50.2)	0.56 (0.41,0.77)***	121 (43.4)	1.76 (1.28,2.42)***	18 (6.5)	1.21 (0.63,2.31)
Incentive+support	137 (52.7)	0.62 (0.45,0.85)**	102 (39.2)	1.48 (1.07,2.06)*	21 (8.1)	1.54 (0.82,2.88)
**Men (N = 360)**						
Control	105 (69.5)	1.00	41 (27.2)	1.00	5 (3.3)	1.00
Incentive only	61 (55.5)	0.55 (0.33,0.91)*	45 (40.9)	1.86 (1.10,3.13)*	4 (3.6)	1.10 (0.29,4.20)
Incentive+support	58 (58.6)	0.62 (0.37,1.05)	36 (36.4)	1.53 (0.89,2.64)	5 (5.1)	1.55 (0.44,5.51)
**Women (N = 568)**						
Control	145 (60.9)	1.00	77 (32.4)	1.00	16 (6.7)	1.00
Incentive only	79 (46.7)	0.56 (0.38,0.84)**	76 (45.0)	1.71 (1.14,2.57)*	14 (8.3)	1.25 (0.60,2.64)
Incentive+support	79 (49.1)	0.62 (0.41,0.93)*	66 (41.0)	1.45 (0.96,2.20)	16 (9.9)	1.53 (0.74,3.16)

*p<0.05;

**p<0.01;

***p<0.001.

Stratification of the stage of readiness outcomes by gender showed that, among men, it was only those in the I-O group who showed significantly different odds ratios relative to the control group of being in the precontemplation stage (OR 0.55, 95% CI 0.33–0.91) and contemplation/preparation stages (OR 1.88, 95% CI 1.10–3.13). This was also the pattern observed among women, with odds ratios of each of these outcomes in the I-O group being 0.56 (95% CI 0.38–0.84) and 1.71 (95% CI 1.14–2.57), respectively. Among women, those in the I+S group were less likely than those in the control group to be in the precontemplation stage for PARC attendance (OR 0.62, 95% CI 0.41–0.93).

### Physical activity participation

After 12 months there remained similar proportions in each group who were very inactive (<20 mins/week of MVPA) at 12 months (Table 4). However, a significantly lower proportion of participants in the I+S group were classified as low active (20–149 mins/week) compared with those in the control group (OR 0.71, 95% CI 0.50–0.99). Although the I+S group had the highest proportion of participants classified as being sufficiently active (≥150 mins/week) at 12 months, the odds of this outcome did not reach significance when compared with the control group. There were no significant differences between the trial groups in the proportions of participants who improved their level of physical activity from baseline to 12 months.

**Table 4:** PA level and improvement over 12 months.

Group	<20 min/wk (very inactive)		20–149 min/wk (low active)		≥150 min/wk (sufficiently active)		Improved MVPA level	
	n (%)	OR (95%CI)	n (%)	OR (95%CI)	n (%)	OR (95%CI)	n (%)	OR (95%CI)
**All (N = 928)**								
Control	127 (32.6)	1.00	140 (36.0)	1.00	122 (31.4)	1.00	109 (28.0)	1.00
Incentive only	91 (32.6)	1.00 (0.72,1.39)	98 (35.1)	0.96 (0.70,1.33)	90 (32.3)	1.04 (0.75,1.45)	77 (27.6)	0.98 (0.70,1.38)
Incentive+support	86 (33.1)	1.02 (0.73,1.42)	74 (28.5)	0.71 (0.50,0.99)*	100 (38.5)	1.37 (0.98,1.90)	83 (31.9)	1.21 (0.86,1.70)
**Men (N = 360)**								
Control	44 (29.1)	1.00	56 (37.1)	1.00	51 (33.8)	1.00	42 (27.8)	1.00
Incentive only	31 (28.2)	0.95 (0.55,1.64)	35 (31.8)	0.79 (0.47,1.33)	44 (40.0)	1.31 (0.79,2.18)	31 (28.2)	1.02 (0.59,1.76)
Incentive+support	32 (32.3)	1.16 (0.67,2.01)	26 (26.3)	0.60 (0.35,1.05)	41 (41.4)	1.39 (0.82,2.34)	38 (38.4)	1.62 (0.94,2.77)
**Women (N = 568)**								
Control	83 (34.9)	1.00	84 (35.3)	1.00	71 (29.8)	1.00	67 (28.2)	1.00
Incentive only	60 (35.5)	1.03 (0.68,1.55)	63 (37.3)	1.09 (0.72,1.64)	46 (27.2)	0.88 (0.57,1.36)	46 (27.2)	0.95 (0.61,1.48)
Incentive+support	54 (33.5)	0.94 (0.62,1.44)	48 (29.8)	0.78 (0.51,1.20)	59 (36.6)	1.36 (0.89,2.08)	45 (28.0)	0.99 (0.63,1.55)

MVPA–moderate and vigorous physical activity.

*p<0.05.

When stratified by gender, the I+S group tended to have the lowest proportion of participants classified as low active and the highest proportion classified as sufficiently active, but the differences between the trial groups were not significant for either men or women. While the highest proportion of men who showed progression in their level of physical activity was in the I+S group, this also did not reach significance compared with the control group.

### Social and cognitive influences upon physical activity

Analysis of PA activity intentions, attitudes, subjective norms, self-efficacy, action planning and anticipated regret showed that these did not differ significantly between the trial groups at baseline and (as shown in Table 5) these similarities remained after 12 months. The lack of difference between the groups was also found when sub-group analysis was undertaken for men and women.

**Table 5:** Social and cognitive determinants of PA at 12 months.

Group	Intention		Attitude		Subjective norm		Action planning		Self-efficacy		Anticipated regret	
	n (%)	OR (95%CI)	n (%)	OR (95%CI)	n (%)	OR (95%CI)	n (%)	OR (95%CI)	n (%)	OR (95%CI)	n (%)	OR (95%CI)
**All (N = 928)**												
Control	318 (81.7)	1.00	306 (78.7)	1.00	356 (91.5)	1.00	227 (58.4)	1.00	344 (88.4)	1.00	335 (86.1)	1.00
Incentive only	230 (82.4)	1.05 (0.70,1.57)	228 (81.7)	1.21 (0.82,1.79)	256 (91.8)	1.03 (0.59,1.80)	167 (59.9)	1.06 (0.78,1.46)	244 (87.5)	0.91 (0.57,1.46)	241 (86.4)	1.02 (0.65,1.60)
Incentive+support	220 (84.6)	1.23 (0.80,1.88)	203 (78.1)	0.97 (0.66,1.41)	245 (94.2)	1.51 (0.81,2.85)	156 (60.0)	1.07 (0.78,1.47)	227 (87.3)	0.90 (0.56,1.45)	221 (85.0)	0.91 (0.59,1.43)
**Men (N = 360)**												
Control	125 (82.8)	1.00	121 (80.1)	1.00	143 (94.7)	1.00	83 (55.0)	1.00	138 (91.4)	1.00	131 (86.8)	1.00
Incentive only	90 (81.8)	0.94 (0.49,1.78)	89 (80.9)	1.05 (0.57,1.96)	99 (90.0)	0.50 (0.20,1.30)	64 (58.2)	1.14 (0.69,1.87)	101 (91.8)	1.06 (0.44,2.57)	94 (85.5)	0.90 (0.44,1.82)
Incentive+support	80 (80.8)	0.88 (0.46,1.69)	76 (76.8)	0.82 (0.44,1.51)	93 (93.9)	0.87 (0.29,2.58)	55 (55.6)	1.02 (0.62,1.71)	85 (85.9)	0.57 (0.26,1.28)	85 (85.9)	0.93 (0.44,1.93)
**Women (N = 568)**												
Control	193 (81.1)	1.00	185 (77.7)	1.00	213 (89.5)	1.00	144 (60.3)	1.00	206 (86.6)	1.00	204 (85.7)	1.00
Incentive only	140 (82.8)	1.13 (0.67,1.88)	139 (81.2)	1.33 (0.81,2.19)	157 (92.9)	1.54 (0.75,3.15)	103 (60.9)	1.02 (0.68,1.53)	143 (84.6)	0.85 (0.49,1.50)	147 (87.0)	1.11 (0.63,1.98)
Incentive+support	140 (87.0)	1.55 (0.89,2.73)	127 (78.9)	1.07 (0.66,1.74)	152 (94.4)	1.98 (0.90,4.37)	101 (62.7)	1.10 (0.73,1.66)	142 (88.2)	1.16 (0.63,2.13)	136 (84.5)	0.91 (0.52,1.59)

## Discussion

Leisure and aquatic centres provide opportunities for both organised and non-organised physical activity in communities. The available evidence suggests that these facilities are a feature of the built environment associated cross-sectionally with exercise and recreational activity among residents, yet there is limited longitudinal research and few intervention studies that have investigated whether they can attract and engage typically inactive people. This population-based RCT found that awareness raising, education, and incentives to promote usage of a new multi-purpose leisure and aquatic centre prompted contemplation and occasional visits by inactive adults in the surrounding municipality over a period of 12 months, but did not influence physical activity levels. This indicates that the strategies tested will not contribute to a reduction in NCD risk, and raises questions about the nature and intensity of interventions required to leverage the opportunities presented by facilities of this type in public health efforts to reduce inactivity.

Among the few studies that have investigated the impact of strategies to promote community-wide usage of leisure and aquatic facilities, those offering entry at nil-cost have shown the greatest impact. An investigation of the *re*:*fresh* scheme that offered free access to leisure centres (swimming pools and gyms) in North West England found, in a time-series study, a 64% increase in gym and swimming activity at these facilities over a 7 year period [27]. Accompanying the fee removal were outreach and health coaching strategies led by locally employed Health Trainers, and a range of promotional activities. In a before-and-after study, the *Gym for Free* scheme in a deprived area in Birmingham, United Kingdom, doubled the proportion of users (from 25% to 64%) who attended most days per week [28]. Regular attendance was incentivised by offering continued free entry to those who used the leisure centres at least 4 times per month, but data were only collected from people attending a facility when the scheme was introduced. A quasi-experimental study investigating the impact of a new multi-purpose aquatic and exercise facility (with accompanying promotions and attendance incentives) in Nagaizumi, Japan, found an increase in users from 275 to 443 person/day over two years, but no difference from the comparison town in the proportion of residents meeting physical activity guidelines [29].

In the present study, two low cost, ‘light touch’ strategies were used to promote PARC usage, comprising PARC information and one free trial pass or, in the expanded intervention with customer relations support, an additional follow-up telephone call and mailed materials. However, the additional follow-up elements within the more intensive intervention were not found to improve the impact achieved by a single invitation letter and free trial pass over 12 months. Notably, the interventions appeared to have no significant impact upon social and cognitive determinants of physical activity (e.g., self-efficacy, attitude, action planning), which suggests that other strategies, potentially involving more direct contacts and/or incentives are necessary to activate these mechanisms of behaviour change. There is a substantial body of evidence that telephone-based advice and counselling is effective in promoting the adoption of physical activity, particularly when this is matched to the readiness, interests and abilities of individuals [30, 31]. Research concerning the impact of mHealth strategies, particularly text messaging, also indicates that these can be used to encourage physical activity participation [32, 33]. It is yet to be determined, however, whether these intervention modalities are effective in promoting attendance at pay-for-use leisure and aquatic facilities among the general population. Given the abovementioned studies [27, 28] that have reported increases in usage of leisure facilities when entry fees were removed, it may be that any behavioural strategies using telephone or mHealth methods need to be accompanied by fee reductions to achieve impacts upon levels of centre usage. Aside from cost, studies among users of public leisure centres indicate that there are other tangible factors of importance for customers, including facility comfort, quality of equipment and overall cleanliness and presentation, that may affect how they value these services [34, 35].

It was notable that a substantial proportion of the inactive people (around three-quarters) recruited into this trial reported having an ongoing health condition, which indicates that family physicians and other health service providers may have a valuable role to play in promoting the use of facilities like PARC by their inactive patients. The exercise referral model, which has been extensively tested in the United Kingdom (UK), entails primary care providers identifying individuals who require the health benefits of physical activity and referring them to a third-party service (e.g., a leisure centre or sports facility) where they receive exercise advice and support [36]. From its review of the evidence, the National Institute for Health and Clinical Excellence has concluded that exercise referral schemes might be beneficial for those who are sedentary or inactive [37]. Schemes of this type have not been tested in Australia, and evidence from the UK indicates that if trialled these should include adequate social support (from clinicians, family and peers, and exercise providers) to facilitate transition and ongoing attendance at the recommended facility [37, 38]. Other important considerations include identifying individuals who are motivated to improve their physical activity, that the exercise facilities are accessible in terms of cost and location, and that the physical activity opportunities are enjoyable and manageable [38, 39].

Strengths of this study were that participants were a cross-sectional sample of typically inactive residents living in the municipality where PARC was introduced, who had not been regular attenders at other gym or aquatic facilities in the preceding year. Thus, we were able to investigate the potential population impacts of the strategies to encourage the use of this Centre. Furthermore, to our knowledge, this is the first randomised trial of strategies to raise awareness and promote attendance at a leisure and aquatic centre. A study limitation was that usage of PARC was measured by self-report, and consequently subject to recall bias, but this bias is likely to have been non-differential across the trial groups. In this study, self-report was necessary because it was not possible to obtain identified admission records for users of PARC who were enrolled in the study. A number of the measures of cognitive and social determinants of physical activity (e.g., self-efficacy, attitudes) were adapted from other scales to improve their suitability and practicality, and the psychometric properties of these modified questions has not been established. While it was possible to examine potential intervention moderation by gender, the relatively low number of participants in the youngest age group (18–34 yrs) provided insufficient power to compare intervention effects across the major categories of age.

## Conclusion

The low intensity interventions trialled in this study were able to prompt improved readiness and occasional use of a new centre by inactive adults, but not regular attendance or increased physical activity. Given the significant costs that are borne by governments to establish leisure and aquatic centres, and the range of physical activity opportunities that these offer, further investigation of strategies to promote their use and health benefits is warranted. Questions remain about the need for more frequent and/or ongoing contact with non-users, potentially incorporating a wider range of communication channels, to improve the contribution of leisure facilities of this type towards physical activity participation and NCD risk reduction. Establishment of referral linkages between health care providers and facility providers could be developed and tested as a means of engaging the many inactive people living with chronic conditions. From a public policy perspective, there are grounds for considering whether financial subsidies can be provided to facility operators, so that fee reductions and/or incentive schemes can be put in place to attract new users. The health and economic benefits of these types of investment will require ongoing evaluation.

## Supporting information

S1 File (PDF)

S1 Text (DOCX)
