# Supplementary material for: Twelve-month findings of the MOVE Frankston randomised controlled trial of interventions to increase recreation facility usage and physical activity among adults
Source: PLoS One. 2021 Jul 23;16(7):e0254216. doi: 10.1371/journal.pone.0254216 (PMC8301672; doi:10.1371/journal.pone.0254216)
Supplement: S1 File — (PDF) [file pone.0254216.s001.pdf]

Trial Review

COVID-19 studies are our top priority. For all other trials, there is a 4-week delay in processing a trial submitted/resubmitted to the ANZCTR and additional delays for updates of registered trials. We appreciate your patience.

The safety and scientific validity of this study is the responsibility of the study sponsor and investigators. Listing a study does not mean it has been endorsed by the ANZCTR. Before participating in a study, talk to your health care provider and refer to this information for consumers

< BACK

Trial registered on ANZCTR

**Registration number**	
**Ethics application status**	Yes
**Date submitted**	10/12/2014
**Date registered**	
**Date last updated**	

Titles & IDs

**Public title**	MOVE Frankston Study
**Scientific title**	Randomised controlled trial of the impact of incentives and support to increase organised physical activity among inactive residents of Frankston
**Secondary ID [1]**	Nil
**Universal Trial Number (UTN)**	U1111-1165-0798
**Trial acronym**	MOVE Frankston
**Linked study record**	

Health condition

Health condition(s) or problem(s) studied:

Physical activity
Mental health

**Condition category**	**Condition code**
Public Health	Health promotion/education
Mental Health	Studies of normal psychology, cognitive function and behaviour

Intervention/exposure

**Study type**	Interventional
**Description of intervention(s) / exposure**	Incentives in the form of trial passes to the Peninsula Aquatic and Recreation Centre, with additional support through telephone contact, SMS reminders and written information about physical activity
**Intervention code [1]**	Behaviour
**Intervention code [2]**	Lifestyle
**Comparator / control treatment**	Control group members receive no treatment.
**Control group**	Active

Outcomes

**Primary outcome [1]**	Physical activity participation
Timepoint [1]	12 month and 24 months after recruitment
**Primary outcome [2]**	Mental well being
Timepoint [2]	12 months and 24 months after recruitment
**Secondary outcome [1]**	Self efficacy
Timepoint [1]	12 months and 24 months after recruitment
**Secondary outcome [2]**	Attitudes
Timepoint [2]	12 months and 24 months after recruitment

Eligibility

**Key inclusion criteria**	Reside in Frankston City Council area; do not meet the Australian national physical activity guidelines; do not attend gym or leisure centre on a weekly basis
**Minimum age**	18 Years
**Maximum age**	70 Years
**Gender**	Both males and females
**Can healthy volunteers participate?**	Yes
**Key exclusion criteria**	Inability to converse in English; physical illness or disability that prevents walking for 10 minutes or more; cognitive problem that impairs communication or reasoning

Study design

**Purpose of the study**	Prevention
**Allocation to intervention**	Randomised controlled trial
**Procedure for enrolling a subject and allocating the treatment (allocation concealment procedures)**	Recruitment and allocation were done by separate researchers. Allocation took place centrally several weeks after recruitment and baseline measurement by means of computer generated random numbers.
**Methods used to generate the sequence in which subjects will be randomised (sequence generation)**	Simple randomisation using a randomised number sequence created by computer software
**Masking / blinding**	Blinded (masking used)
**Who is / are masked / blinded?**	
	The people assessing the outcomes
**Intervention assignment**	Factorial
**Other design features**	
**Phase**	Phase 2
**Type of endpoint(s)**	Efficacy
**Statistical methods / analysis**	Sample size was determined as the number needed to show a significant difference in weekly participation in organised physical activity. It is assumed that 10% of control subjects will become regular participants in organised physical activity. Therefore a sample size of 300 in each arm of the study will be required to show with 95% confidence limits and 80% power: (i) a 10% difference in outcome between participants in Intervention 1 and the control group (i.e., 20% vs. 10%); and (ii) a 10% difference between those in Intervention 1 and Intervention 2 (i.e., 30% vs. 20%). To accommodate a 25% drop out among recruited participants over the two year course of the study, the target sample size was inflated to 400 per group.

Recruitment

**Recruitment status**	Closed: follow-up continuing

Date of first participant enrolment

**Anticipated**		**Actual**	24/07/2014

Date of last participant enrolment

**Anticipated**		**Actual**	17/09/2014

Date of last data collection

**Anticipated**		**Actual**	

Sample size

Target	1200	Accrual to date	Final

Recruitment in Australia

**Recruitment state(s)**	VIC
**Recruitment postcode(s) [1]**	3195 - Aspendale
**Recruitment postcode(s) [2]**	3196 - Chelsea
**Recruitment postcode(s) [3]**	3197 - Carrum
**Recruitment postcode(s) [4]**	3198 - Seaford
**Recruitment postcode(s) [5]**	3199 - Frankston
**Recruitment postcode(s) [6]**	3200 - Frankston North
**Recruitment postcode(s) [7]**	3201 - Carrum Downs
**Recruitment postcode(s) [8]**	3911 - Langwarrin South
**Recruitment postcode(s) [9]**	3910 - Langwarrin
**Recruitment postcode(s) [10]**	3977 - Cranbourne

Funding & Sponsors

**Funding source category [1]**	Government body
**Name [1]**	Australian Research Council
**Address [1]**	GPO Box 2702<br>CANBERRA<br>ACT 2601
**Country [1]**	Australia
**Funding source category [2]**	Government body
**Name [2]**	Frankston City Council
**Address [2]**	30 Davey Street,<br>Frankston VIC 3199
**Country [2]**	Australia
**Primary sponsor type**	University
**Name**	Monash University
**Address**	Research Office<br>Monash University<br>Building 3d<br>Monash University<br>Victoria 3800
**Country**	Australia
**Secondary sponsor category [1]**	None
**Name [1]**	
**Address [1]**	
**Country [1]**	

Ethics approval

**Ethics application status**	Yes
**Ethics committee name [1]**	Monash University Human Reseach Ethics Committee
**Ethics committee address [1]**	First Floor, Building 3e<br>Room 111<br>Monash Research Office<br>Clayton Campus<br>Monash University VIC 3800
**Ethics committee country [1]**	Australia
**Date submitted for ethics approval [1]**	
**Approval date [1]**	26/05/2014
**Ethics approval number [1]**	CF14/1148 - 2014000497

Summary

Brief summary

Physical inactivity is prevalent in Australia and is a ignificant contributor to the nation's burden of chronic disease. Increasing the availability of recreational infrastructure could, however, positively influence levels

of physical activity. This study, conducted in partnership with Frankston City Council, will investigate the impact that establishing a major recreational and aquatic centre has upon physical activity and measures of well being in the community. It will test the additional impacts of using marketing strategies to boost centre usage, and thereby inform future action to multiply the health benefits of public investments in recreational infrastructure.

Trial website www.move-frankston.org.au

Trial related presentations / publications

Public notes

Contacts

Principal investigator

Name A/Prof Ben Smith

Address School of Public Health and Preventive Medicine
Monash University
Level 6
99 Commercial Rd
Melbourne, Victoria, 3004

Country Australia

Phone +61 3 99031654

Fax

Email ben.smith@monash.edu

Contact person for public queries

Name A/Prof Ben Smith

Address School of Public Health and Preventive Medicine
Monash University
Level 6
99 Commercial Rd
Melbourne, Victoria, 3004

Country Australia

Phone +61 3 99031654

Fax

Email ben.smith@monash.edu

Contact person for scientific queries

Name A/Prof Ben Smith

Address School of Public Health and Preventive Medicine
Monash University
Level 6
99 Commercial Rd
Melbourne, Victoria, 3004

Country Australia

Phone +61 3 99031654

Fax

Email ben.smith@monash.edu

No information has been provided regarding IPD availability

Summary results

No Results

< BACK
