# Supplementary material for: Twelve-month findings of the MOVE Frankston randomised controlled trial of interventions to increase recreation facility usage and physical activity among adults
Source: PLoS One. 2021 Jul 23;16(7):e0254216. doi: 10.1371/journal.pone.0254216 (PMC8301672; doi:10.1371/journal.pone.0254216)
Supplement: S1 Text — (DOCX) [file pone.0254216.s002.docx]

Access to the MOVE Frankston trial dataset is available from DOI: 10.26180/14559462.
